# Netrin-1 Stimulates Migration of Neogenin Expressing Aggressive Melanoma Cells

**DOI:** 10.3390/ijms232112751

**Published:** 2022-10-22

**Authors:** Gustavo Untiveros, Aleksandr Raskind, Laura Linares, Alessandro Dotti, Luigi Strizzi

**Affiliations:** 1 Department of Pathology, College of Graduate Studies, Midwestern University, Downers Grove, IL 60515, USA; 2 Chicago College of Osteopathic Medicine, Midwestern University, Downers Grove, IL 60515, USA

**Keywords:** Netrin-1, Neogenin, migration, aggressive melanoma cells

## Abstract

Netrin-1 is a neural guidance factor that regulates migration and positioning of neural crest-derived cells during embryonic development. Depending on the type of Netrin-1 receptor expression, cells are either attracted or repulsed by Netrin-1. Postnatal expression of Netrin-1 is detected in brain, colon, liver, and kidney, which are common sites of cancer metastasis, including melanoma. Thus, understanding the dynamics between Netrin-1 and its receptors could explain the attraction of melanoma towards these Netrin-1-expressing tissues. Here, we investigate whether the Netrin-1-attractive receptor Neogenin can affect migration of melanoma cells towards a Netrin-1 source. Results from Western blot (WB) analysis show higher expression of Neogenin in aggressive compared to non-aggressive melanoma cells. Cell migration experiments show increased migration of Neogenin-expressing aggressive melanoma cells towards exogenous, soluble recombinant human Netrin-1 and towards a Netrin-1-expressing cell line. Furthermore, WB reveals ERK1/2 activation and increased N-cadherin expression in Neogenin-expressing aggressive melanoma cells treated with rhNetrin-1. Moreover, treatment with anti-Neogenin blocking antibody caused decreased migration towards Netrin-1-expressing cells and reduced ERK1/2 activity in Neogenin-expressing aggressive melanoma cells. These results suggest Neogenin may play a role during migration of melanoma cells towards Netrin-1 via ERK1/2 signaling.

## 1. Introduction

For 2022, the American Cancer Society estimates 99,780 diagnoses of melanoma in the United States, and of those patients, approximately 7650 will succumb to the disease [1]. Cutaneous melanoma arises from the malignant transformation of melanocytes, the pigment producing cells of the skin [2]. Despite melanoma being the least common among skin cancers, it is the most aggressive and prone to metastasis [2]. Melanoma is a multifactorial disease that can be caused by both environmental and genetic factors [3,4]. A significant environmental risk factor is exposure to ultraviolet radiation associated with increased outdoor activity or frequent use of indoor tanning facilities [5,6,7]. In terms of susceptibility, individuals with certain phenotypical features, such as fair skin and red hair, are at higher risk for melanoma compared to people with increased skin pigmentation [8]. Melanin has been found to have protective abilities not only by physically obstructing UV radiation, thereby reducing the amount of rays capable of reaching the cell nucleus and causing DNA damage, but also reducing the effects of oxidative stress by acting as a scavenger of radical oxygen species [9,10]. When DNA damage occurs, this can lead to mutations that alter tumor-suppressor gene function and/or activate select oncogenes that lead to uncontrolled proliferation, cell survival, and migration [11]. 

Neurulation is a complex embryonic developmental process that gives rise to the formation of the neural plate and other structures that ultimately differentiate into components of the nervous system, including the brain and spinal cord [12]. In this process, morphogenic and signaling proteins, electrochemical gradients, and neural growth factors play important roles during proliferation and migration of neural cells towards their final destinations [13]. Netrin-1 is a laminin-like protein that can act as a chemoattractant or chemorepellent of neuronal cell migration and axonal extension depending on the type of receptor expressed by the target cell [14]. For instance, cells that express the Netrin-1 receptors DCC (deleted in colon cancer) or Neogenin are attracted to Netrin-1 [14]. In contrast, cells that express the family of UNC (uncoordinated) receptors, such as UNC5, are repulsed by Netrin-1 [14]. Furthermore, binding of Netrin-1 to UNC5 and DCC also appears to regulate cell survival [15]. Expression of Netrin-1 and its receptors has also been found play a role in tumorigenesis in several human cancers, such as breast and colon cancer [16]. However, conflicting data have emerged regarding the expression and role of Netrin-1 and its receptors in melanoma. For instance, one study showed that melanoma cells do not express Netrin-1 or DCC but express UNC5 receptors, which trigger endothelial cell repulsion [17]. That same study also showed expression of Neogenin receptors in the melanoma cells analyzed, but its role was not clarified. In contrast, another report described expression of both Netrin-1 and DCC in melanoma, which appeared to play a role in survival and progression of melanoma [18]. Results from another study suggest that expression of Netrin-1 receptors in melanoma is irrelevant and that only upregulation of Netrin-1 is needed to increase cell motility [19], yet another study showed that Netrin-1 actually inhibited the mobility of melanoma cells [20]. Reports of Netrin-1 and Neogenin in migratory and invasive processes of other neoplasias have also revealed complex downstream signaling activation [21,22,23], further confounding our understanding of the precise role of these molecules in melanoma progression. Treatments for melanoma currently involve surgical excision of lesions, traditional chemotherapy, therapy targeting specific signaling pathways, and approaches that use different variations of immune therapy [24]. However, high rates of disease relapse and metastatic spread often lead to failure of these therapies. Once melanoma has metastasized, the 5-year survival rate decreases to less than 30% [25]. Therefore, early detection and better treatment strategies are imperative for improving patient survival. 

The goal of this research is to investigate the role of neural guidance molecules in melanoma progression. Few studies have examined the mechanism that enables certain melanoma cells to have a propensity to metastasize for specific sites. Melanocytes are derived from the neural crest and are influenced by the same neural crest-associated factors that regulate growth, differentiation, and migration of cells during neurulation; therefore, we hypothesize that these same factors could play a role during the progression and metastatic spread of melanoma. Since there is a scarcity of information regarding Netrin-1/Neogenin interaction in melanoma, the aim of this study is to determine if Neogenin receptor expression in melanoma cells can facilitate migration towards Netrin-1. 

## 2. Results

### 2.1. Neogenin Expression Is Greater in Aggressive vs. Poorly Aggressive Melanoma Cells

**Figure 1:**
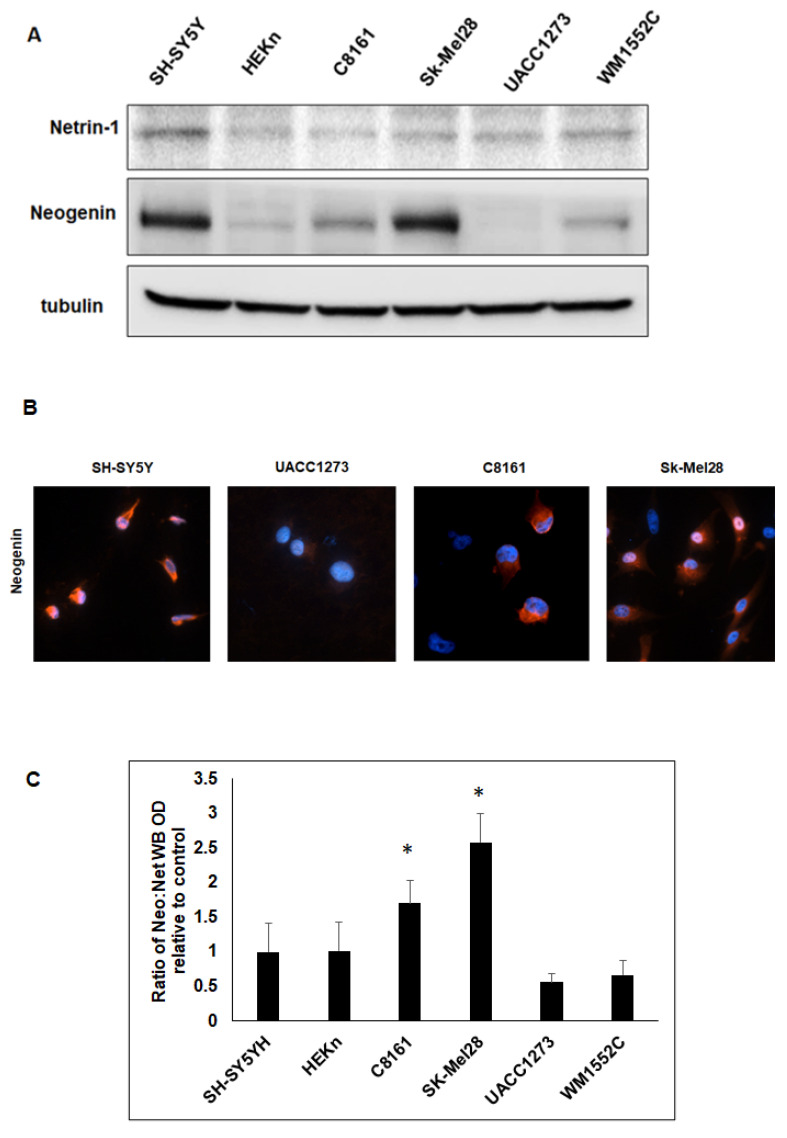
Expression of Netrin-1 and Neogenin in human melanoma cells. (**A**) Western blot results show different levels of Netrin-1 and Neogenin expression in human melanoma cell lines. The human neuroblastoma cell line SH-SY5Y and the normal human epidermal keratinocyte cell line HEKn were used as positive-expressing and poorly expressing controls for cellular expression of Netrin-1, respectively. (**B**) Results from immunofluorescence (IF) staining shows more intense expression of Neogenin in the aggressive melanoma cell lines C8161 and Sk-Mel28 compared to the poorly aggressive melanoma cell line UACC1273. SH-SY5Y was used as positive control for the IF staining for Neogenin. (Original magnification 20X). (**C**) The histogram shows the ratio of Neogenin to Netrin-1 expression determined from densitometric analysis of the respective WB bands and represented as a fold difference relative to the Neogenin-to-Netrin-1 ratio calculated for SH-SY5Y, which was used as control. These results clearly show that Neogenin expression relative to Netrin-1 is significantly greater in aggressive compared to poorly aggressive melanoma cells (* *p* < 0.05, *n* = 3).

Western blot analysis was used to determine baseline expression of Netrin-1 and Neogenin in the melanoma cell lines C8161, Sk-Mel28, UACC1273, and WM1552C. The neuroblastoma cell line SH-SY5Y and the human normal keratinocyte cell line HEKn were used, respectively, as high and poor cellular expression controls for Netrin-1 [26,27]. WB results show variable expression of Netrin-1 in the different melanoma cell lines (Figure 1A). However, Neogenin expression was greater in the aggressive melanoma cell lines C8161 and Sk-Mel28 compared to the poorly aggressive melanoma cell lines UACC1273 and WM1552C. Results from immunofluorescence staining confirmed the enhanced expression of Neogenin in aggressive melanoma cells (C8161 and Sk-Mel28) compared to poorly aggressive melanoma cells (UACC1273) (Figure 1B). Since previous studies have linked receptor density to ligand sensitivity [28,29], we proceeded to compare the ratio of Neogenin to Netrin-1 expression, calculated from the WB OD values obtained above, for the different cell lines analyzed to determine whether melanoma cells with excess Neogenin expression may have the potential for increased sensitivity to Netrin-1. Using the ratio of Neogenin to Netrin-1 expression for SH-SY5Y as a reference control (1.0 +/− 0.41), we found that the Neogenin-to-Netrin-1 ratio for C8161 and Sk-Mel28 was, respectively, 1.70 +/− 0.33- and 2.58 +/− 0.42-fold of control and significantly greater than the Neogenin-to-Netrin-1 ratio for UACC1552C and WM1552C, which was, respectively, 0.56 +/− 0.13- and 0.67 +/− 0.21-fold of control (Figure 1C, *p* < 0.05). These results show that although Netrin-1 is variably expressed in the different cell lines tested, the Neogenin-to-Netrin-1 ratio expression is much higher in aggressive versus poorly aggressive melanoma cells, suggesting that excess Neogenin could increase sensitivity of aggressive melanoma cells to Netrin-1.

### 2.2. Netrin-1 Acts as a Chemoattractant for Aggressive Melanoma Cells

**Figure 2:**
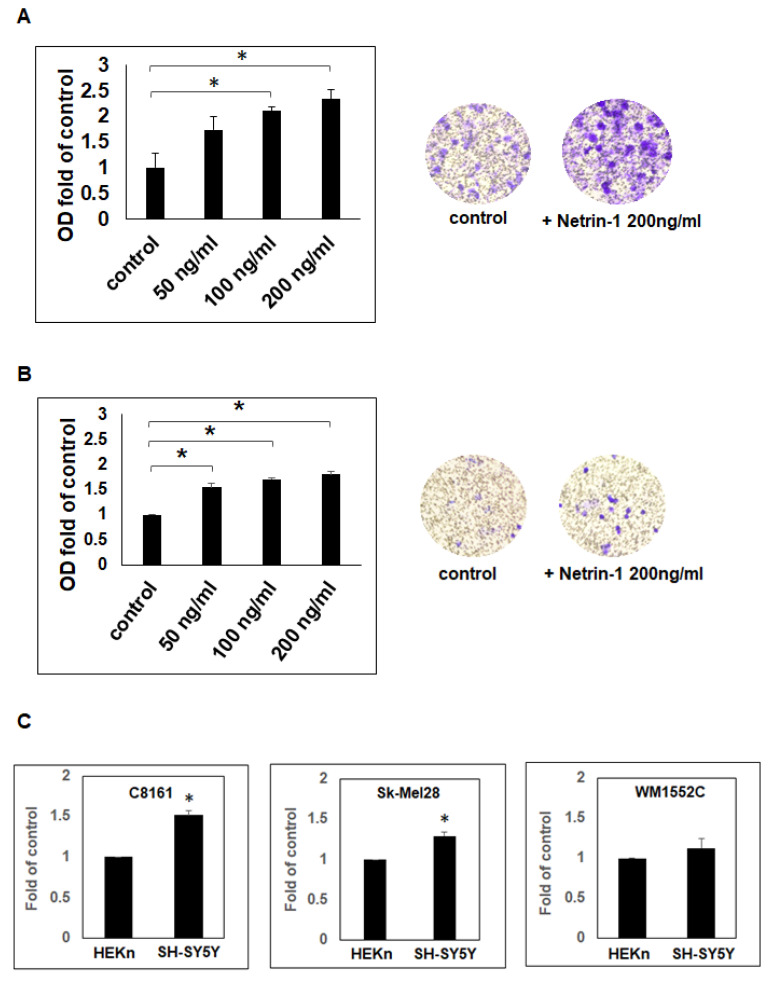
Netrin-1 is a chemoattractant for C8161 and Sk-Mel28 aggressive melanoma cells. Results from cell migration assay show that the treatment with exogenous, soluble rhNetrin-1 at 50, 100, and 200 ng/mL is associated with a dose-dependent increase in migration of (**A**) C8161 (*n* = 3) and (**B**) Sk-Mel28 (*n* = 2) aggressive melanoma cells (* *p* < 0.05) compared to control. Representative microphotographic images at 10x magnification show increased numbers of the migrated crystal violet-stained C8161 (**A**) and Sk-Mel28 (**B**) aggressive melanoma cells towards 200 ng/mL of rhNetrin-1. (**C**) Further results from cell migration assays show significantly greater migration of C8161 (*n* = 3) and Sk-Mel28 (*n* = 3) aggressive melanoma cells towards the Netrin-1-expressing SH-SY5Y cells compared to migration towards the poorly-Netrin-1-expressing HEKn cells (* *p* < 0.05). In contrast, there was no significant difference in migration of the low-Neogenin, poorly aggressive WM1552C melanoma cells (*n* = 3) towards SH-SY5Y compared to HEKn cells.

Since Neogenin expression was shown to be higher in aggressive compared to poorly aggressive melanoma cells and that the ratio of Neogenin to Netrin-1 expression is also much greater for the aggressive compared to the poorly aggressive melanoma cells, we sought to determine whether the excess expression of Neogenin would translate in greater responsiveness to Netrin-1 in the aggressive melanoma cells. C8161 and Sk-Mel28 aggressive melanoma cells were seeded in a standard transwell cell migration chamber and treated with increasing concentrations (50 ng/mL, 100 ng/mL, and 200 ng/mL) of exogenous, soluble rhNetrin-1. Migration of C8161 showed a significant, dose-dependent increase in migration in the presence of 100 ng/mL and 200 ng/mL of rhNetrin-1, which was, respectively, 2.1 +/− 0.07- and 2.3 +/− 0.19-fold of control (Figure 2A, *p* < 0.05). Migration of Sk-Mel28 also showed a significant, dose-dependent increase in migration in the presence of 50 ng/mL, 100 ng/mL, and 200 ng/mL of rhNetrin-1 that was, respectively, 1.5 +/− 0.08-, 1.7 +/− 0.03-, and 1.8 +/− 0.06-fold of control (Figure 2B, *p* < 0.05). There was no statistical significance in migration between the different rhNetrin-1 treatment groups for both C8161 and Sk-Mel28 aggressive melanoma cells. In contrast, migration of the poorly aggressive melanoma cell line WM1552C, which expressed low levels of Neogenin receptor as determined by WB analysis, did not show any significant migratory difference in the presence of exogenous, soluble rhNetrin-1 compared to control. These results demonstrate that Neogenin receptors expressing aggressive melanoma cells are attracted towards exogenous, soluble rhNetrin-1. 

We conducted additional experiments to assess whether Neogenin receptor expressing melanoma cells are attracted towards a cellular source of Netrin-1. To this end, we performed a migration assay with the Neogenin receptor expressing C8161 and Sk-Mel28 aggressive melanoma cells that were incubated in the presence of the Netrin-1-expressing SH-SY5Y cells or poor-Netrin-1-expressing HEKn human keratinocytes. Results show that migration of the C8161 and Sk-Mel28 aggressive melanoma cells towards SH-SY5Y is significantly greater at, respectively, 1.5 +/− 0.06- and 1.29 +/− 0.05-fold of migration towards HEKn cells, used as control (Figure 2C, *p* < 0.05). Meanwhile, migration of the poorly aggressive, low-Neogenin-expressing melanoma cell line WM1552C showed no significant difference in migration towards SH-SY5Y compared to HEKn cells (Figure 2C). These results demonstrate that Neogenin-expressing aggressive melanoma cells are attracted towards Netrin-1-expressing cells.

### 2.3. Netrin-1 Can Stimulate ERK1/2 Activity and Increase Expression of N-Cadherin in Aggressive Melanoma Cells

**Figure 3:**
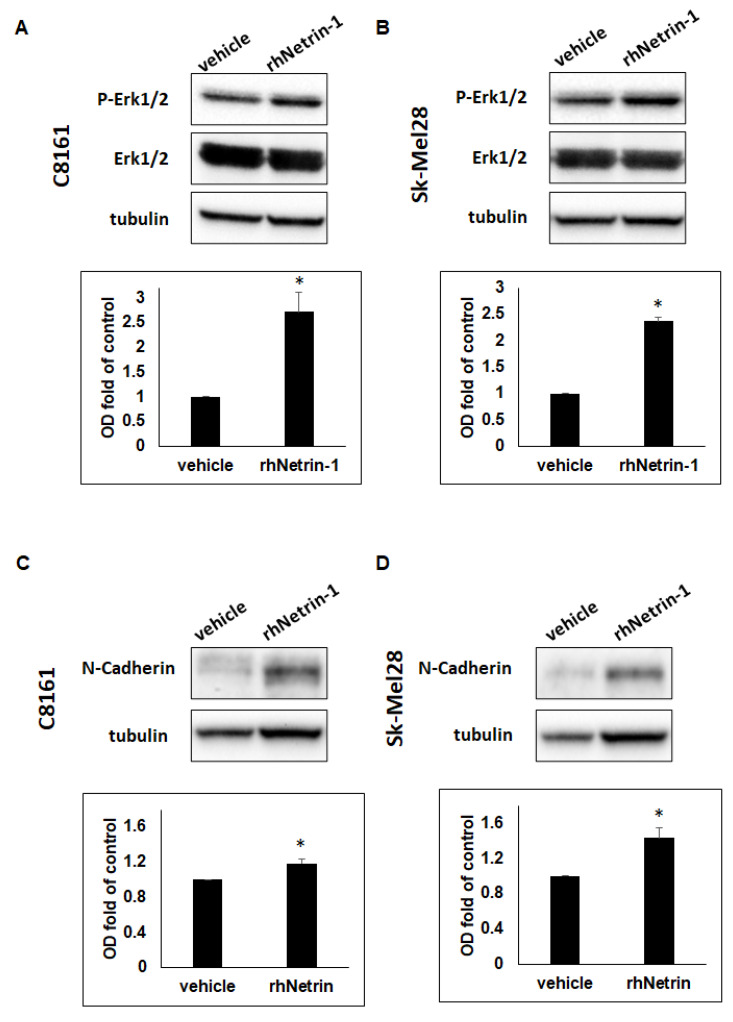
Netrin-1 stimulates pERK1/2 activation and N-cadherin expression in aggressive melanoma cells. Results from WB show significantly increased expression of P-ERK1/2 (**A**,**B**) and N-cadherin (**C**,**D**) in C8161 (*n* = 3) and Sk-Mel28 (*n* = 3) aggressive melanoma cells treated with 200 ng/mL of exogenous, soluble rhNetrin-1, compared to vehicle control (* *p* < 0.05).

Existing reports indicate that Netrin-1 can activate ERK1/2 signaling in some types of cancer [30,31]. To elucidate the molecular mechanism through which the interaction of Netrin-1 and Neogenin receptors regulates migration, we performed WB analysis on the Neogenin receptor expressing aggressive melanoma cells C8161 and Sk-Mel28 treated with exogenous, soluble rhNetrin-1 and probed for pERK1/2. Both C8161 and Sk-Mel28 aggressive melanoma cells experienced a significant increase in pERK1/2 levels that was, respectively, 2.72 +/− 0.381- and 2.37 +/− 0.075-fold of control after treatment with 200 ng/mL of exogenous, soluble rhNetrin-1 (Figure 3A,B, *p* < 0.05). To further substantiate the effect of Netrin-1 on enhancing the migration of aggressive melanoma cells, we also probed the cell lysates from the rhNetrin-1-treated C8161 and Sk-Mel-28 for the pro-migratory/pro-metastatic molecule N-cadherin [32]. Our results show that treatment of C8161 and Sk-Mel28 aggressive melanoma cells with 200 ng/mL of rhNetrin-1 was associated with a significant increase in N-cadherin expression (C8161: 1.19 +/− 0.05-fold of control; Sk-Mel-28: 1.43 +/− 0.12-fold of control) (Figure 3C,D, *p* < 0.05). 

### 2.4. Neogenin Blocking Antibodies Significantly Reduces Migration of Neogenin Receptor Expressing C8161 and Sk-Mel28 Cells towards Netrin-1-Expressing Cells and ERK1/2 Activity

**Figure 4:**
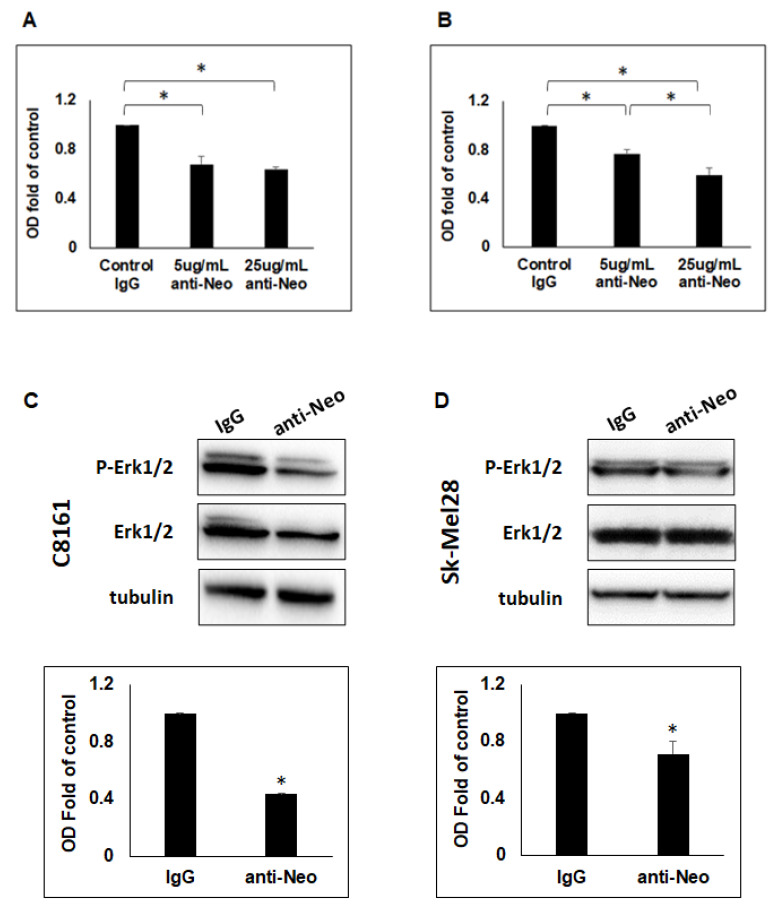
Effect of anti-Neogenin function blocking antibody on migration of aggressive melanoma cells towards a cellular source of Netrin-1 and ERK1/2 activity. Results of cell migration assays for (**A**) C8161 (*n* = 3) and (**B**) Sk-Mel28 (*n* = 3) aggressive melanoma cells towards Netrin-1-expressing SH-SY5Y cells was significantly reduced after treatment with either 5 µg/mL or 25 µg/mL of anti-Neogenin blocking antibody for both cell lines when compared to control IgG. Migration of Sk-Mel28 was also significantly lower when treated with 25 µg/mL compared to 5 µg/mL of anti-Neogenin blocking antibody (* *p* < 0.05). Additional WB analysis showed a significant decrease of P-ERK1/2 in (**C**) C8161 (*n* = 3) and (**D**) Sk-Mel28 (*n* = 3) aggressive melanoma cells treated with 5 µg/mL or 25 µg/mL, respectively, of anti-Neogenin blocking antibody compared to IgG control (* *p* < 0.05).

To determine whether the increased migration of the Neogenin receptor expressing aggressive melanoma cells C8161 and Sk-Mel28 was due to Netrin-1 and Neogenin receptor interaction, we conducted a migration assay using C8161 or Sk-Mel28 aggressive melanoma cells that were incubated in the presence of SH-SY5Y and treated with either 5 µg/mL or 25 µg/mL of anti-Neogenin blocking antibody. Compared to cells treated with IgG control, we observed a significant decrease in migration of C8161 cells (0.68 +/− 0.07 and 0.64 +/− 0.02-fold of control) and Sk-Mel28 cells (0.76 +/− 0.04 and 0.59 +/− 0.06-fold of control) when treated with 5 µg/mL or 25 µg/mL of anti-Neogenin blocking antibody, respectively (Figure 4A,B, *p* < 0.05). In addition, although there was no statistical significance between the different anti-Neogenin treatment groups for C8161, migration of Sk-Mel28 aggressive melanoma cells towards the Netrin-1-expressing SH-SY5Y was significantly lower when treated with 25 μg/mL compared to 5 μg/mL of anti-Neogenin (Figure 4B, *p* < 0.05). Furthermore, we demonstrated a significant decrease in pERK1/2 levels in C8161 aggressive melanoma cells treated with 5 μg/mL of anti-Neogenin blocking antibody (0.43 +/− 0.01-fold of control) and in Sk-Mel28 aggressive melanoma cells treated with 25ug/mL of anti-Neogenin blocking antibody (0.65 +/− 0.11-fold of control) (Figure 4C,D, *p* < 0.05). These results demonstrate that the Neogenin receptor plays a role in the migration of C8161 and Sk-Mel28 cells towards a cellular source of Netrin-1 and that ERK1/2 signaling is potentially involved in this function.

## 3. Discussion

Netrin-1 is a guidance molecule that plays an important role during development mainly by regulating migration and positioning of neural crest-derived cells and directing axonal pathways [14]. Depending on the receptor type, cells are either attracted towards or repulsed away from Netrin-1. Classic Netrin-1-attractive receptors are DCC and Neogenin, while Netrin-1-repulsive receptors generally belong to the UNC family of receptors [14]. Netrin-1 and its receptors have also been shown to play a role in neuronal plasticity that is involved in learning, memory [33], and in survival and progression of non-neuronal cells, most importantly cancer cells of the breast and colon [34]. Few studies have shown expression of these molecules in melanoma [17,18,19,20]; however, it is not clear from these reports what precise role Netrin-1 and its receptors play during melanoma progression. 

In our study, we initially assessed Netrin-1 expression in melanoma cell lines. We confirm that Netrin-1 can be detected by WB analysis in melanoma; however, we could not find significant differences in Netrin-1 expression between the aggressive and poorly aggressive melanoma cells studied. We also evaluated the expression of Neogenin receptor expression by WB analysis and found that the aggressive melanoma cell lines C8161 and Sk-Mel28 showed higher Neogenin expression compared to the poorly aggressive melanoma cell lines UACC1273 and WM1552C. These results are particularly intriguing, as, firstly, to our knowledge, there are no clear data on the difference of Neogenin receptor expression between aggressive and poorly aggressive melanoma cells. Secondly, since Neogenin receptor plays a role in attracting cells towards Netrin-1, this suggests that Neogenin-receptor-positive melanoma cells could have a migratory advantage towards Netrin-1-expressing tissues during metastatic spread. In fact, we demonstrated that the Neogenin receptors expressing aggressive melanoma cells, C8161 and Sk-Mel28, which also showed a significantly greater ratio of Neogenin to Netrin-1 expression, demonstrated significantly increased migration towards exogenous, soluble rhNetrin-1 and, most importantly, towards Netrin-1-expressing cells compared to control. Complementary to these results, treatment of C8161 and Sk-Mel28 aggressive melanoma cells with different concentrations of function-blocking anti-Neogenin antibody significantly reduced the migration of these cells towards the Netrin-1-expressing cells. 

Neogenin-regulated cell activity is highly variable and dependent on cell type and functional context. A previous study showed that presenilin-1-associated gamma-secretase activity localized to lipid raft complexes can trigger release of the Neogenin intracytoplasmic domain, which presumably leads to increased aggressiveness in ganglioside-positive melanoma cells, suggesting that presenilin-1 may regulate Neogenin-dependent melanoma aggressiveness [35]. However, this appears in contrast with a more recent study that showed how advanced-stage/aggressive melanoma actually expresses low levels of presenilin-1 and that gamma-secretase inhibition in aggressive melanoma cells increased presenilin-1 levels and reduced melanoma cell migration [36]. Here, we show that Netrin-1/Neogenin interaction in aggressive melanoma cells leads to ERK1/2 activation and increased expression of N-cadherin. These data add molecular support to the role of Netrin-1 in enhancing migration of aggressive melanoma cells. This is of particular interest given the involvement of ERK1/2 in proliferation, survival, and cell migration in multiple other neoplasias [23,30,31] and the pro-migratory and pro-metastatic effects of N-cadherin in cancer [32] and of its association with ERK1/2 activity [37,38,39]. Overall, our results support the role of Netrin-1/Neogenin interaction during the migration of melanoma cells towards Netrin-1 and that ERK1/2 signaling and N-cadherin expression are potentially involved in this migratory activity. 

Up to 60% of patients with advanced-stage melanoma are diagnosed with brain metastasis [40]. Moreover, brain metastases have been detected at autopsy in over 70% of patients who died from metastatic melanoma [41]. Therefore, there is a need to better understand what drives the high propensity for brain metastasis in melanoma. Our data suggest that Neogenin receptors could play an important role during the metastatic spread of melanoma towards Netrin-1-expressing tissues such as the brain. Interestingly, studies have shown that Alzheimer’s disease, a neurodegenerative process characterized by accumulation of amyloid beta in brain tissue, which results in functional/cognitive decline, is associated with poor Netrin-1 expression [42,43,44]. This is not surprising given the contribution of Netrin-1 to synaptic function, which is involved in learning and memory [33,45]. One way that Netrin-1 may protect against Alzheimer’s is by negatively regulating amyloid peptide [46]. Since Alzheimer’s patients appear to have reduced melanoma incidence [47], the low Netrin-1 expression and high amyloid beta levels produced in Alzheimer’s brains could represent a possible protective factor against melanoma. In contrast, the relatively higher levels of Netrin-1 in brains of non-Alzheimer’s patients could facilitate the formation of metastatic melanoma lesions by attracting, as suggested by our data, Neogenin-receptor-expressing aggressive melanoma cells. 

In summary, the results from our study provide the scientific rationale for exploring novel therapeutic approaches in targeting neuronal guidance factor/receptor interactions, such as Netrin-1/Neogenin, as a means to reduce disease progression and improve patient survival in melanoma.

## 4. Materials and Methods

### 4.1. Cell Cultures

The human melanoma cell lines WM1552C (Rockland Immunochemicals, Limerick, PA, USA, WM1552C-01-0001), C8161 and UACC1273 (gifts from Dr. Richard Seftor, University of West Virginia), and Sk-Mel28 (ATCC, Manassas, VA, USA, HTB-72) were used. These cells were maintained in RPMI1640 media supplemented with 5% HI-FBS for WM1552C (RPMI: GenClone, San Diego, CA, USA, 25-506H; FBS: Seradigm, Batavia, IL, USA, 97068-085), RPMI1640 media supplemented with 10% HI-FBS for C8161, EMEM (ATCC, 30-2003) with supplemented 10% HI-FBS for Sk-Mel28, and RPMI1640 supplemented with 10% HI-FBS and 0.1% Gentammycin for UACC1273. We also used the primary cell line epidermal keratinocytes (HEKn) (ATCC, PCS-200-010) that were grown in Dermal Cell Basal Media (ATCC, PCS-200-030) and keratinocyte growth supplement (ATCC, PCS-200-040) and the Neuroblastoma cells SH-SY5Y (ATCC, CRL-2266) that were grown in EMEM/F12 containing 10% HI-FBS (EMEM: ATCC 30-2003; F12: Gibco, Waltham, MA, USA, 11765-054). The SH-SY5Y neuroblastoma cells were used as a Netrin-1-expressing positive control, while the HEKn epidermal keratinocytes were used as a poor-Netrin-1-expressing control. Cells were incubated in 37 °C and 5% CO_2_ conditions.

### 4.2. Cell Treatments

C8161, Sk-Mel28, or WM1552C melanoma cells were treated with varied concentrations of exogenous, soluble recombinant human Netrin-1 (rhNetrin-1) (R&D Systems, Minneapolis, MN, USA, 6419-N1-025) with a PBS vehicle control or with an anti-Neogenin blocking antibody (R&D, AF1079) with a Goat IgG control (Millipore, Burlington, MA, USA, I5256-10MG) for either migration assays or Western blotting (WB). For our dose-dependent migration assay, we treated C8161, Sk-Mel28, and WM1552C cells with 50, 100, and 200 ng/mL of soluble rhNetrin-1 and 200 ng/mL for the migration treatment of the representative images (see section below). We also used 5 and 25 µg/mL of the anti-Neogenin antibody in our migration assay setup for C8161 and Sk-Mel28 cells grown in the presence of SH-SY5Y cells (see section below). For WB, we treated the aggressive melanoma cells C8161 and Sk-Mel28, seeded in a 6-well plate, with 200 ng/mL of rhNetrin-1 or PBS vehicle control for 6 h; we also treated C861 and Sk-mel28 cells, respectively, with 5 µg/mL or 25 µg/mL of the anti-Neogenin blocking antibody and control IgG overnight.

### 4.3. Protein Lysis, Extraction, and Western Blot

For cell lysis and protein extraction, we grew C8161, Sk-Mel28, WM1552C, UACC1273, SH-SY5Y, and HEKn cells in T-75 flasks or C8161 and Sk-Mel28 cells grown in 6-well plates for treatment setups, as stated above. Cells were collected, washed, and lysed using standard RIPA buffer (Pierce, Waltham, MA, USA) with phosphatase and protease inhibitors (Pierce). Protein lysates were then collected and spun at 16,000g for 30 min at 4 °C. For WB, SDS-PAGE was performed using a 4–20% gradient gel (Novex), and 30 µg of protein were loaded per well. Protein was then transferred to a PVDF membrane (Millipore), washed thrice in TBST, and then blocked for 1 h at RT in blocking buffer. The membranes were incubated with appropriate dilutions of primary antibody in blocking buffer overnight at 4 °C. The following antibodies were used: anti-Neogenin (R&D, AF1079, 1:500 in 5% non-fat dry milk (NFDM)), anti-Netrin-1 (R&D, AF6419, 1:500 in 5% bovine serum albumin (BSA)), anti-α-tubulin (Cell Signaling, Danvers, MA, USA, 2144s, 1:1000 in 5% NFDM), anti-pERK1/2 (Cell Signaling, 9101, 1:1000 in 5% BSA), anti-ERK1/2 (Cell Signaling, 4695, 1:1000 in 5% NFDM), and anti-N-cadherin (Cell Signaling, 13116, 1:1000 in 5% BSA). Next, the membranes were washed with TBST and then incubated with an appropriate dilution of conjugated secondary antibody in blocking buffer for 1 h at RT. Secondary antibodies were anti-goat (R&D, HAF109, 1:5000 in 5% NFDM), anti-rabbit (GE Amersham, Marlborough, MA, USA, NA934, 1:5000 in 5% NFDM), and anti-sheep (R&D, HAF016, 1:5000 in 5% (BSA). Membranes were washed with TBST and incubated in ECL (Amersham), and images were obtained using the Bio-Rad Universal hood III Chemidoc system. For quantification of WB results, densitometric analysis of WB bands was performed using the ImageJ analysis software. The resulting optical density (OD) values are reported as OD signals normalized to OD values of alpha-tubulin loading control. For phosphorylated proteins, results are presented as the ratio of phosphorylated protein to total protein. 

### 4.4. Immunofluorescence Staining

The human melanoma cells C8161, Sk-Mel28, and UACC1273 and the neuroblastoma SH-SY5Y cell were seeded on glass coverslips in a 24-well plate at 100,000 cells/well. Cells were washed once with cold PBS and then fixed in ice cold 2% PFA in PBS for 20 min. Cells were then washed thrice with cold PBS and then blocked for 1 h with 2% BSA/PBS at 4 °C with rocking. For antibody detection, we used a Rabbit anti-Neogenin antibody at 1 µg/mL (Novus, Centennial, CO, USA, NBP1-89651) diluted in 2%BSA/PBS, and for negative control, we used Rabbit IgG (Vector Laboratories, Newark, CA, USA, I-1000) which was used at 1 µg/mL diluted in 2%BSA/PBS; all were incubated overnight at 4 °C with rocking. Coverslips were washed thrice in PBS at 4 °C with rocking. Conjugated Alexafluor-594 anti-rabbit secondary antibody (Jackson ImmunoResearch, West Grove, PA, USA, 711-586-152) was added at 1:500 in 2%BSA/PBS. Coverslips were washed thrice in PBS at 4 °C with rocking, rinsed with cold MilliQ, and then mounted on slides with DAPI-hardset mounting media (Vector Laboratories, H-1800). 

### 4.5. Migration Assays

The human melanoma cells C8161 and Sk-Mel28, WM1552C, neuroblastoma SH-SY5Y cells, and primary keratinocytes HEKn were grown as in the conditions stated above. All three migration assay setups were performed on 3 µm pore transwells for 24-well plates; C8161, Sk-Mel28, or WM1552C cells were seeded at a density of 150,000 cells per transwell, and cell migration was incubated for 16 h at 37 °C and 5% CO_2_ conditions for all setups. Our first assay involved C8161-, Sk-Mel28-, or WM1552C-seeded transwells in 24-well plates and treated with media containing variable concentrations of exogenous, soluble rhNetrin-1 or PBS vehicle control, as stated above. Our second migration assay involved C8161-, Sk-Mel28-, or WM1552C-seeded transwells in 24-well plates and incubated in the presence of SH-SY5Y or HEKn cells. Our final assay involved C8161- or Sk-Mel28-seeded transwells in 24-well plates and incubated in the presence of SH-SY5Y cells while being treated with two different concentrations of anti-Neogenin blocking antibody or Goat IgG control, as stated above. After 16 h incubation, the medium was removed, the outside of the transwells were washed with PBS, and a cotton swab was moistened and flattened with PBS to gently swab the non-migrated cells from the interior of the transwell and place them in fresh well of PBS, repeated once. Transwells were then transferred to a 24-well plate with crystal violet solution and incubated on a rocking platform for 10 min at RT. Transwells were then washed with PBS and transferred to a 24-well plate with 10% acetic acid solution for 30 min at RT. Extracted stain from each sample was transferred to a 96-well plate, and absorbance was read at 590nm. To obtain representative images of cell migration, cells were handled as stated above; however, cells were fixed in methanol for 20 min at RT before staining with crystal violet solution for 30 s. Cells were washed with PBS, and the growth surface (bottom of transwell) was cut out and mounted on a slide using Cytoseal-60 (Thermo Scientific, Waltham, MA, USA, 23-244257) and then observed and photographed under light microscopy.

### 4.6. Statistical Analysis

For statistical analysis, GraphPad statistical software was used to perform *t*-tests comparing the mean values calculated from the results of at least two independent experiments, each performed in triplicate (+/− standard error of the mean (SEM)), between experimental and control. For experiments involving three or more independent conditions, one-way ANOVA with post hoc Tukey analysis was performed to determine statistical significance between the different groups. Analysis generating a *p*-value of less than 0.05 was considered statistically significant. 

## Data Availability

Data are contained within the article. The data presented in this study are available on request from the corresponding author.
